# A Review of Cerebral Amyloid Angiopathy, Its Overlap With Alzheimer's Disease and Its Implications on a Memory Service

**DOI:** 10.1192/bjo.2026.11586

**Published:** 2026-06-30

**Authors:** Alexander Sunderland, Ruth Kinnear, Christopher Raj, Lucy Hickling, Tarun Kuruvilla

**Affiliations:** Gloucestershire Health and Care NHS Foundation Trust, Gloucester, United Kingdom

## Abstract

**Aims:**

Cerebral Amyloid Angiopathy (CAA) is a type of vascular disease present in more than 80% of confirmed Alzheimer’s disease patients. It is characterized by the presence of Amyloid-*β* protein deposited within the walls of vessels which results in various clinical presentations including vascular rupture of fragile amyloid-β laden vessels. Over the past 5 years, there has been a reported significant increase in publication, diagnoses, and referrals of CAA**.**

**Methods:**

In our memory service, features of CAA including peripheral microhaemorrhages and superficial siderosis were reported in 28% of MRI’s requested in the past year, with CAA specifically mentioned in 50% of those. This therefore poses several challenges on a memory service regarding how these should be interpreted and how they may affect the patient’s management plan, including how the patients stroke primary prevention should be reviewed in this setting, which will be outlined in this poster.

**Results:**

CAA and Alzheimer’s disease (AD) frequently co-occur due to their shared proteinopathy and both diseases share impaired clearance through perivascular drainage pathways as a primary pathogenic mechanism. However, they differ in location of protein deposition and peptide composition. Their clinical overlap is critical for memory services, as CAA accelerates cognitive decline through mechanisms such as white matter structural disconnection and microinfarction, even in the absence of acute haemorrhage.

The emergence of anti-Amyloid monoclonal antibodies also makes CAA awareness mandatory. These treatments can trigger Amyloid-Related Imaging Abnormalities (ARIA), which represent an iatrogenic form of CAA-related inflammation. Pre-treatment screening with MRI is essential and current guidelines suggest that patients with features of CAA including microbleeds and cortical superficial siderosis should generally be excluded from these AD therapies due to high ARIA risk.

For patients diagnosed with CAA, aggressive vascular risk factor control is paramount to reduce recurrent haemorrhage risk. In patients with probable CAA, clinicians should exercise caution with anticoagulants, statins, and regular NSAID use, as these may exacerbate haemorrhagic risk in high-burden, and antiplatelet treatment for primary prevention is not recommended. This suggests improved collaboration between stroke and cognitive specialists is necessary to manage the complex balance between ischaemic prevention and haemorrhagic risk.

**Conclusion:**

Increasing awareness of CAA among doctors, including within memory services, is vital for accurate diagnosis, risk stratification, and the safe implementation of emerging AD therapeutics. This poster outlines the current evidence on the pathophysiology of CAA, including its overlap and differences with AD, and its current and future implications focussing on a memory service.

